# Psychosomatic consultation in the workplace: do we reach different users by changing the context?

**DOI:** 10.1186/1472-6963-14-S2-P105

**Published:** 2014-07-07

**Authors:** Eva Rothermund, Reinhold Kilian, Michael Hoelzer, Monika Annemarie Rieger, Harald Guendel

**Affiliations:** 1 University Clinic of Psychosomatic Medicine and Psychotherapy, Ulm, Germany; 2 Klinik für Psychiatrie II, Ulm/Guenzburg, Germany; 3 Sonnenbergklinik Stuttgart, ZfP, Stuttgart, Germany; 4 Institut für Arbeitsmedizin, Sozialmedizin, Versorgungsforschung Universität Tuebingen, Tuebingen, Germany

## Background

In Germany, the proportion of mental health diagnoses in early retirement is currently at 40%, constituting the largest diagnostic group. Due to demographic changes, shortage of qualified staff is an increasing challenge for social insurance funds and the labour market. Work-related stress is known to promote common mental disorders (CMD) like depression, anxiety, functional somatoform disorder or adjustment disorders and results in a declining quality of life and work performance. Although the landscape of mental health services is well established in Germany, only 40% of affected individuals managed to obtain professional care. The unmet need for easily accessible and early interventions and the economic impact of CMD led to the development of a variety of offers at the interface between company supported and conventional mental health care (CAU) e.g. the “psychosomatic consultation in the workplace” (PCIW). To learn more about this complex system we set out to analyse user profile and change 12-weeks after consultation.

## Materials and methods

Observational cross-sectional design, followed by a pre-post- test 12 weeks after initial consultation. By latent class analysis (LCA) individuals were classified into distinct groups based on individual response patterns. For pre-post comparison we performed variance analysis with repeated measurements. Data were collected by self-administered questionnaires: work ability (work ability index, WAI), quality of life (SF-12), mental health (PHQ9-depression, PHQ-15-somatization, PHQ-7-anxiety) and work-related stress (irritation scale, maslach burnout inventory).

## Results

Preliminary sample description: N=352 individuals: PCIW n=173 /CAU n=179. Demographic variables that differed between the groups (p< 0.05) were age in years (PCI W44.9, SD10.1 /CAU 39.4, SD11.9), gender (PCIW 70% male/CAU 30% male), symptom duration in months (median PCIW 12 /CAU 24) and service utilization, i.e. previous contact with the psychotherapeutic-psychosomatic-psychiatric health care system (PCIW 38%/CAU 63%). A 4-class solution was chosen due to best fit indices. Four subgroups (classes) of users with different patterns of impairment were identified; generally those with less impairment were seen in the vocational context. Pre-post test showed improvement in both groups for WAI (PCIW 30.25, SD 8.25; CAU 26.57 SD 8.86), depression, anxiety, SF-12 mental health and irritation. There was no difference between groups (PCIW vs. CAU), nor a group time effect.

## Conclusion

PCIW stands for an easy accessible therapeutic offer in the vocational context. Our data suggest that we reach a different type of user. Even though the user profile differs the effect of the *intervention* seems to be **similar.**

